# Anticancer effect of histone deacetylase inhibitor scriptaid as a single agent for hepatocellular carcinoma

**DOI:** 10.1042/BSR20180360

**Published:** 2018-07-13

**Authors:** Linlin Liu, Xiaoyang Sun, Yu Xie, Yinping Zhuang, Ruosi Yao, Kai Xu

**Affiliations:** 1 College of Medical Imaging, Xuzhou Medical University, Xuzhou, Jiangsu, China; 2 Blood Diseases Institute, Xuzhou Medical University, Xuzhou, Jiangsu, China

**Keywords:** apoptosis, hepatocelluar carcinoma, proliferation, Scriptaid

## Abstract

Recurrence is one of the major causes of poor prognosis for patients with hepatocellular carcinoma (HCC), and drug resistance is closely associated with disease recurrence. Histone deacetylase (HDAC) inhibitor scriptaid functions as an anticancer agent in many different types of tumors, but its possible roles in HCC progression have not been explored to date. Herein, we show that HDAC inhibitor scriptaid decreases HCC cell proliferation and induces cell cycle G_2_/M-phase arrest in a dose-dependent manner. Furthermore, scriptaid triggered HCC cell death via transcriptional activation of p21 and subsequent elevated global H3Ac levels. Importantly, we found that scriptaid showed robust antitumor activity against HCC. Thus, our findings indicate that HDAC inhibitor scriptaid could be an important potential candidate for treatment of HCC patients.

## Introduction

Hepatocellular carcinoma (HCC) is one of the most universal cancers around the world [1]. Metastasis and recurrence are the major limiting factors that decrease the average survival rate of 5 years in HCC [2]. Despite great success in diagnosis and therapy in HCC, it is still associated with an adverse prognosis. Therefore, it is urgent to pursue new molecular targetted drugs for effective treatment of HCC and explore the fundamental pathogenesis of HCC.

Histone deacetylases (HDACs) are usually dysregulated in cancers, especially in HCC [3,4]. Recent studies have shown that the expression of both HDAC1 and HDAC2 are positively associated with the mortality of 156 Southeast Asian patients with HCC [5]. Depletion of HDAC1 and HDAC2 increased cell death and inhibited cell viability in hepatocellular cancer cell lines [5]. Additionally, *miR-376a* regulated the epigenetic modification via targetting HDAC9 in HCC, and HDAC9 inhibited *miR-376a* by reducing the H3K18Ac modification levels [6]. Down-regulation of HDAC5 also inhibited liver cancer cell proliferation through mediating cell-cycle arrest and apoptosis [7]. Therefore, targetting HDACs is the most efficient approach to explore the association between HCC and the imbalance of histone acetylation and deacetylation.

Currently, several HDAC inhibitors are being used in tumor therapy or fundamental research. Our previous study showed that HDACi NaBut-induced multiple myeloma cell-cycle G_2_/M-phase arrest and cell apoptosis [8]. Vorinostat treatment led to HCC cell apoptosis via activating caspase-3 [9]. Despite increased numbers of HDAC inhibitors, only resminostat and belinostat have undergone Phase I and II clinical trials for HCCs [10,11]. Novel HDAC inhibitor scriptaid (6-(1,3-dioxo-1H-benzo[*de*]isoquinolin-2-yl)*N*-hydroxyhexanamide) was first identified from high-throughput system screening [12], and it has been reported that scriptaid exhibited its antitumor activities against endometrial, ovarian, colon, and lung cancers, as well as glioma and multiple myeloma [13–17]. In 2006, Lai et al. [18] reported that human sulphatase 1 enzyme enhanced scriptaid-mediated proapoptotic properties in HCC. Nevertheless, scriptaid’s specific function in HCC was unclear.

In the present study, we discovered that HDAC inhibitor scriptaid decreased HCC cell-line viabilities in dose- and time-dependent manner. Furthermore, we also found that scriptaid led to HCC cell-cycle G_2_/M-phase arrest. Scriptaid treatment triggered HCC cell apoptosis via transcriptional activation of p21. Importantly, we found that scriptaid treatment evidently reduced the weights and volumes of HCC cell-line HepG2 primary tumors. These data provide an insight into the therapeutic intervention of advanced HCC.

## Materials and methods

### Cell culture

HepG2 and Hep3B cell lines were purchased from the American Type Culture Collection (ATCC). HCC cell lines were cultured in Dulbecco’s modified Eagle’s medium (DMEM) supplemented with 10% FBS at 37°C with 5% CO_2_.

### Western blot

Experiments were carried out as previously reported [19]. The following antibodies were used against β-actin (Santa Cruz Biotechnology): caspase-3 and caspase-9 (Cell Signaling Technology), PARP1, p21, and H3 (Proteintech), and H3Ac and H4Ac (Active Motif).

### Cell viability analysis

For cell viability testing, 1 × 10^4^ cells/well were seeded into 96-well cell-culture plates. Then, the cells were treated with different doses of scriptaid for 24 h or treated with 5 μM scriptaid for different times. Cell viability was measured using the Cell Counting Kit-8 (CCK-8) assay as previously described [8].

### Cell cycle and apoptosis analysis

The cells were treated using different doses of scriptaid for 24 h. Then, cell cycle distribution was performed as previously reported [8]. The indicated cells treated by scriptaid underwent an apoptosis assay with the Annexin V-FITC/PI Apoptosis Detection kit (KeyGen, China).

### Real-time PCR analysis

Total RNA was isolated from the indicated cells treated with scriptaid using TRIzol reagent (TaKaRa, Japan) according to the manufacturer’s protocol. Then, the RNA was reverse transcribed with reverse transcriptase (Promega). Real-time PCR was performed with the SYBR Green reaction mix and a Roche 480 PCR machine.

### Luciferase reporter gene assay

The indicated cells were seeded in 24-well plates at approximately 30% density. Then, the p21 luciferase reporter gene was transfected into the indicated cells using PolyJet DNA transfection reagent according to the protocol. After 6 h, scriptaid was added into the corresponding wells for 24 h, and then, the luciferase activity was measured as previously described [8].

### Xenograft tumor model

Briefly, BALB/c nude mice were subcutaneously injected with HepG2 cells. Then, DMSO or scriptaid was injected every 2 days, using a dose of 5 mg/kg body weight. After 4 weeks, all mice were killed, and the tumor weights and sizes were measured. The tumor volume was calculated with the formula: V = (length)^2^ × (width)/2. This animal study was approved by the Animal Care Committee of Xuzhou Medical University, China.

### Statistical analysis

All data are presented as the mean ± S.D. of three independent experiments. Student’s *t* test was used to determine the statistical difference. *P*<0.05 was considered as statistically significant. All statistical analyses were carried out using GraphPad Prism 5 software.

## Results

### Scriptaid inhibited HCC cell proliferation

**Figure 1:**
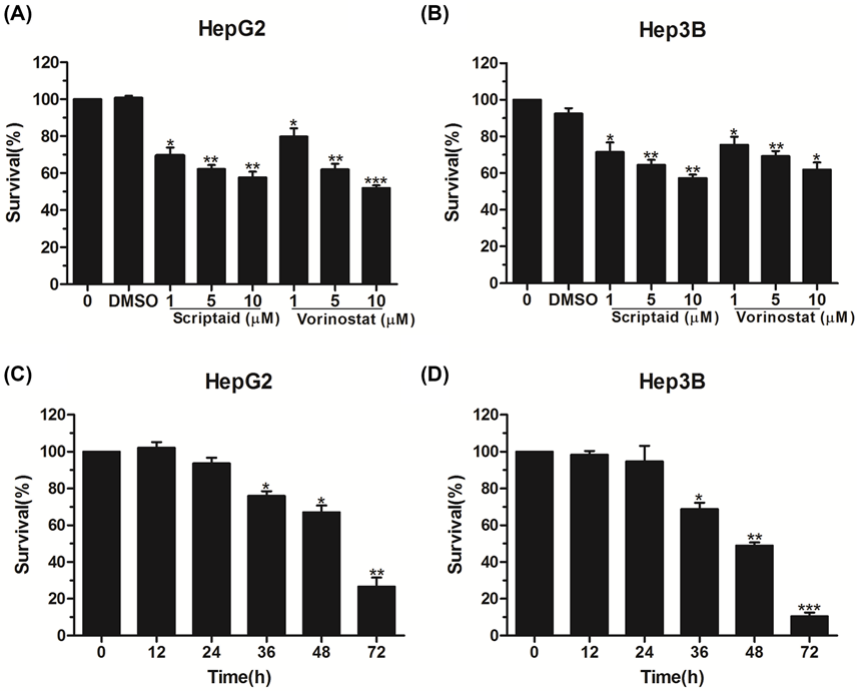
Scriptaid inhibited HCC cell proliferation (**A**,**B**) Cell proliferation analysis of HepG2 and Hep3B cells treated by scriptaid and vorinostat at different concentrations (0, 1, 5, and 10 μM), with DMSO as negative control. (**C**,**D**) CCK-8 analysis of HepG2 and Hep3B cells exposed to 5 μM scriptaid at different times. The data represent three independent experiments. Error bars: mean + S.D.; *, *P*<0.05; **, *P*<0.01; ***, *P*<0.001.

To determine the effect of HDAC inhibitor scriptaid on HCC cell proliferation, HepG2 cells were treated using different concentrations of scriptaid (0, 1, 5, and 10 μM). After 24 h, the CCK-8 assay was conducted, and the results showed that scriptaid inhibited HepG2 cell proliferation in a dose-dependent manner (Figure 1A). Subsequently, we conducted a cell viability assay using another HCC cell line, Hep3B. Carcinoma cell viability was inhibited following treatment with scriptaid in a concentration-dependent manner (Figure 1B). We also compared the antitumor activity between scriptaid and well-known HDAC inhibitor vorinostat, and both of them showed similar antiproliferation activity, implying that scriptaid could be a useful addition to the treatment options for patients with HCC (Figure 1A,B). Additionally, HepG2 and Hep3B cells were also treated with 5 μM scriptaid for different amounts of time, and the results demonstrated that scriptaid visibly decreased multiple HCC cell viabilities in a time-dependent manner (Figure 1C,D). The above data indicate that HDAC inhibitor scriptaid inhibited HCC cell proliferation in a dose- and time-dependent manner.

### Scriptaid induced G_2_/M-phase arrest of HCC cells

**Figure 2:**
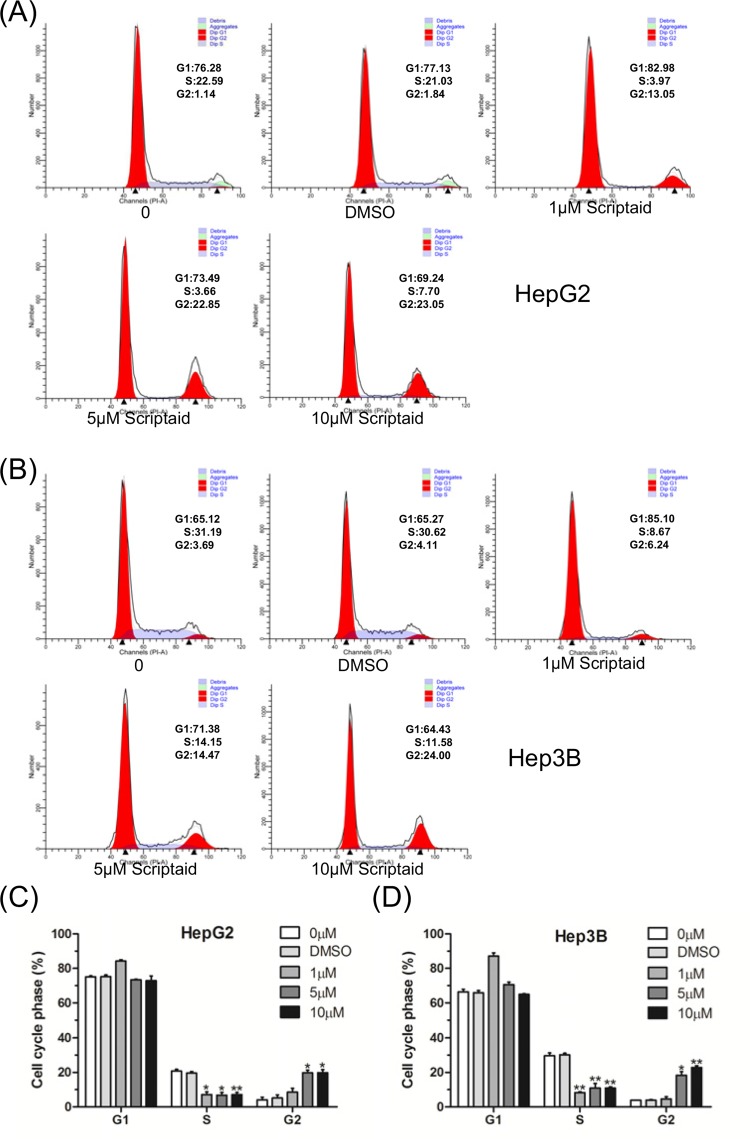
Scriptaid induced G_2_/M-phase arrest of HCC cells (**A**,**B**) HepG2 and Hep3B cells were treated with different concentrations of scriptaid for 24 h, and the cell cycle was detected by flow cytometry. (**C**,**D**) The distribution of the HepG2 and Hep3B cells’ subpopulations treated with scriptaid was analyzed based on three independent experiments. The experiments were conducted three times. Error bars: mean + S.D.; *, *P*<0.05; **, *P*<0.01.

Next, we examined the effect of scriptaid on the cell cycle distribution of HepG2 cells. Following treatment with different concentrations of scriptaid, we found that scriptaid clearly and dose-dependently increased the cell ratio of G_2_/M-phase compared with the DMSO control group (Figure 2A,C). Furthermore, we also detected the effect of scriptaid on the Hep3B cell cycle, and the results indicated that scriptaid treatment led to Hep3B cell cycle G_2_/M-phase arrest in liver cancer cells in a dose-dependent manner (Figure 2B,D).

### Scriptaid promoted apoptosis in HCC cells

**Figure 3:**
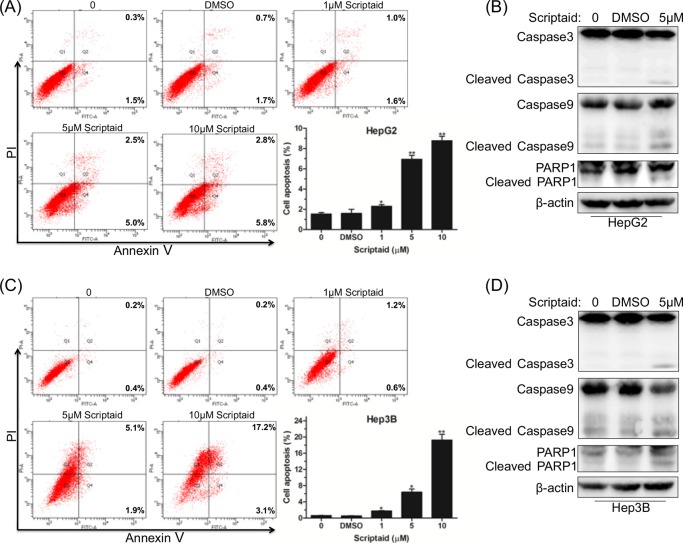
Scriptaid promoted apoptosis in HCC cells (**A**) After treatment with scriptaid for 24 h, HepG2 cells were analyzed using an Annexin V-FITC/PI detection kit. (**B**) Western blot analysis of apoptosis-associated factors caspase-3, caspase-9, PARP1, and their cleaved forms. (**C**) Flow cytometry analyses of Hep3B cell apoptosis following treatment with different doses of scriptaid for 24 h. (**D**) Assessment of caspase-3, caspase-9, PARP1, and corresponding cleaved forms in Hep3B cells treated with scriptaid. Experiments were repeated for at least three times. Error bars: mean + S.D. *, *P*<0.05; **, *P*<0.01.

To clarify the mechanism of scriptaid-induced cytotoxicity in HCC cells, we conducted flow cytometry analysis based on Annexin V-FITC/7-AAD staining. As shown in Figure 3A, scriptaid treatment clearly increased the percentage of apoptotic HepG2 cells in a dose-dependent manner. The caspase family plays an important role in the progression of apoptosis, and therefore, we detected the expression level of apoptosis-associated proteins. The results showed that caspase-3, caspase-9, and PARP1 were activated by proteolytic cleavage in response to scriptaid treatment (Figure 3B). Furthermore, we also found that scriptaid dose-dependently induced Hep3B cell apoptosis (Figure 3C). Western blot results showed elevated expression of activated caspase-3, caspase-9, and PARP1 in Hep3B cell lines (Figure 3D). These data indicated that scriptaid promoted HCC cell apoptosis via activation of caspase-3, caspase-9, and PARP1.

### Scriptaid-induced cell apoptosis was associated with p21 expression

**Figure 4:**
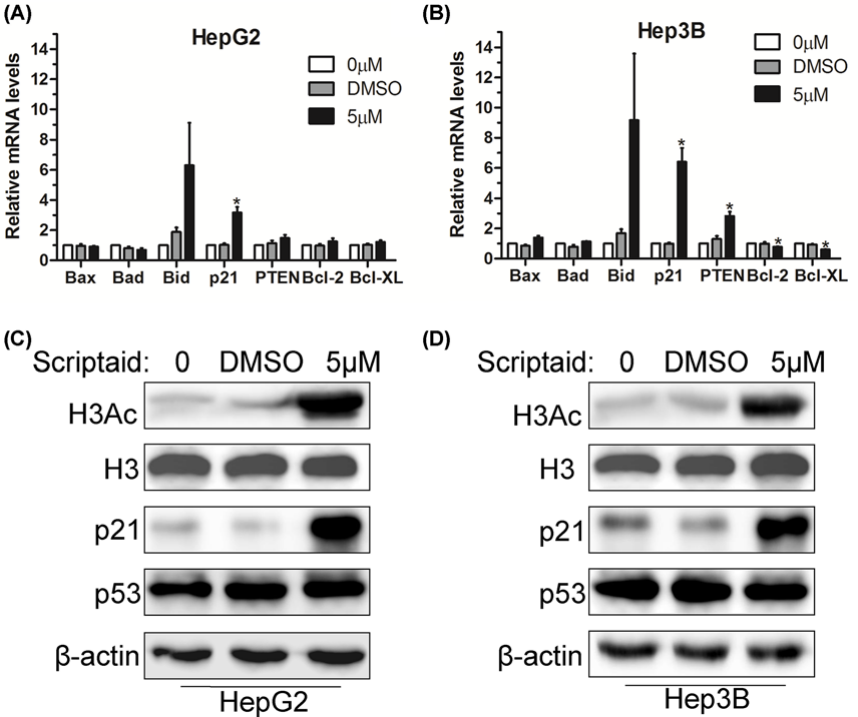
Scriptaid-induced cell apoptosis was associated with p21 expression (**A**,**B**) HepG2 and Hep3B cells were treated with 5 μM scriptaid for 24 h. Expression levels of apoptosis-related factors were determined by Q-PCR. *, *P*<0.05. (**C**,**D**) Western blot analysis of H3Ac, p21, and p53 in HepG2 and Hep3B cells treated with scriptaid. H3 and β-actin were used as internal controls.

To further elucidate the mechanism by which scriptaid inhibited HCC cell proliferation and induced cell apoptosis, we used Q-PCR analysis to identify possible potential targets of scriptaid. We first investigated the expression of various apoptosis- and proliferation-associated factors, and the results indicated that scriptaid treatment clearly increased p21 expression at mRNA levels in HepG2 cells compared with the DMSO control group (Figure 4A). Then, Hep3B cells were also treated with 5 μM scriptaid for 24 h, and Q-PCR analysis showed that it augmented the expression of pro-apoptotic factors p21 and PTEN, with a concomitant decrease in pro-survival factors Bcl-2 and Bcl-XL at mRNA levels (Figure 4B). Therefore, we speculated that p21 could be a key regulator of scriptaid-mediated cell death. However, we found that scriptaid treatment led to a significant decrease in p53 at mRNA levels (data not shown). Specifically, HepG2 and Hep3B cells treated with scriptaid were characterized by a significant increase in p21 expression, but basically no significant change in p53 at protein levels (Figure 4C,D). As an HDAC inhibitor, gene transcription regulation by scriptaid may rely on the changes in H3Ac and H4Ac modification levels. Western blot showed that scriptaid treatment led to a significant increase in the global H3Ac modification levels in both HepG2 and Hep3B cells (Figure 4C,D). Our data confirmed that scriptaid-induced HCC cell apoptosis was associated with p21 expression.

### Antitumor activity of scriptaid in an HCC xenograft model

**Figure 5:**
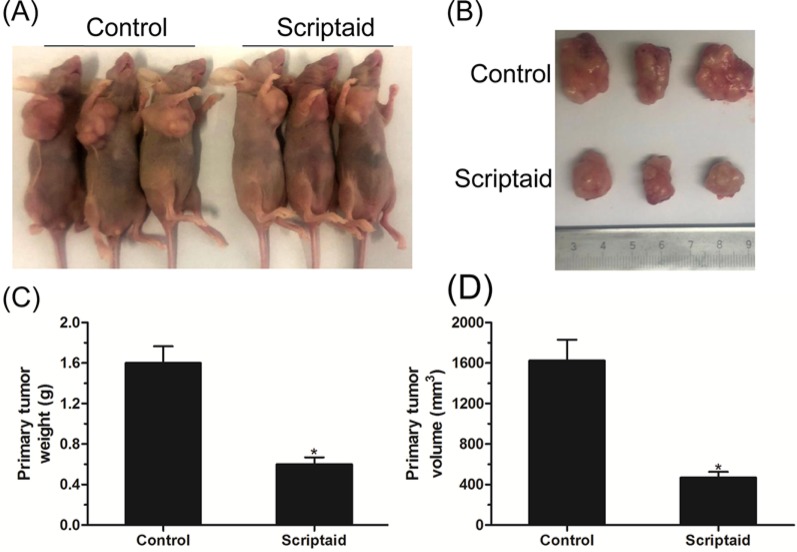
Antitumor activity of scriptaid in an HCC xenograft model (**A**,**B**) Representative image of xenograft tumors from BALB/c nude mice subcutaneously injected with HepG2 cells and treated with PBS or scriptaid twice a week. (**C**,**D**) Primary tumor weights and volumes in BALB/c nude mice that received scriptaid treatment. Error bars: mean + S.D. (*n*=6). *, *P*<0.05.

To explore whether scriptaid had an impact on tumorigenesis *in vivo*, we assessed its ability to repress tumor growth *in vivo* by using a subcutaneous HepG2 murine xenograft model. As shown in Figure 5A,B, scriptaid treatment evidently reduced the tumor growth compared with the untreated group. After 4 weeks, the mice were killed, and the tumor weight and volume were recorded. We detected a marked decrease in the primary tumor weight and volume in mice treated with scriptaid (Figure 5C,D). Collectively, the above data provide evidence for the possibility of clinical trials and treating HCC patients with the HDAC inhibitor scriptaid.

## Discussion

HCC is one of the most common malignancies of primary liver cancer, which leads to a lower patient survival rate because of its metastasis and recurrence. Drug resistance is a major cause for recurrence, and therefore, it is urgent to develop new molecular-targetted therapeutic drugs. Epigenetic regulation is closely associated with HCC progression [20]. Amongst them, histone acetylation and deacetylation are dynamic changes, which require histone acetyltransferase (HAT) and HDAC to mediate gene activation or repression [21]. The imbalance between HAT and HDAC is associated with malignant disease and tumors [22].

HDAC inhibitors can be applied in tumor therapy for various cancers by altering the HDAC expression or disrupting acetylation homeostasis. In recent years, an increasing number of HDAC inhibitors have appeared and served as potential drugs for patients with HCC, such as resminostat, quisinostat, entinostat, and valproic acid [10,23–25]. However, only resminostat underwent a Phase II clinical trial for HCC patients. Therefore, it is still urgent to explore novel HDAC inhibitors and their mechanism of antitumor activities for HCC. In the present study, we found that the novel HDAC inhibitor scriptaid inhibited multiple HCC cell proliferation in a dose- and time-dependent manner. Further study confirmed that scriptaid led to liver cancer cell cycle G_2_/M phase arrest and triggered cell apoptosis.

In terms of the mechanism, we found that scriptaid promoted p21 gene transcription in liver cancer cells, indicating that p21 could be a key regulator of scriptaid-mediated cell apoptosis. It has been reported that p21 interacts with p53 [26]. Surprisingly, tumor suppressor p53 was down-regulated in a manner that corresponded with scriptaid treatment (data not shown). However, the p53 protein levels remained basically unchanged (Figure 4). Therefore, we speculated that scriptaid-induced HCC cell apoptosis was associated with p21 expression, and p21 participated in the scriptaid-mediated antitumor activity independent of p53.

In conclusion, our results proved that HDAC inhibitor scriptaid decreased HCC cell survival and induced cell cycle G_2_/M-phase arrest. p21 could be an important mediator of scriptaid-induced HCC cell death and antitumor activity. Therefore, our study highlights scriptaid’s therapeutic potential.

## Abbreviations

7-AAD: 7-amino actinomycin D
Bcl-2: B cell lymphoma 2
Bcl-xL: B cell lymphoma-extra large
CCK-8: cell counting kit-8
HAT: histone acetyltransferase
HCC: hepatocellular carcinoma
HDAC: histone deacetylase
HDACi: histone deacetylase inhibitor
H3Ac: histone H3 acetylation
H3K18Ac: histone H3 lysine 18 acetylation
PARP1: poly (ADP-ribose) polymerase 1
PTEN: phosphatase and tensin homolog
Q-PCR: quantitative polymerase chain reaction
